# Supplementary material for: Chromothripsis during telomere crisis is independent of NHEJ, and consistent with a replicative origin
Source: Genome Res. 2019 May;29(5):737–49. doi: 10.1101/gr.240705.118 (PMC6499312; doi:10.1101/gr.240705.118)
Supplement: Supplemental Material [file supp_gr.240705.118_Supplemental_file_1.zip › contigs/annotated_contigs/DB51/contig.2.DB51_length_396_mean_cov_3.4797979798.docx]

**DB51_length_396_mean_cov_3.4797979798**

```
ACCAGCAGTGAGATTCTTCACAGAAAGAGATAGATCCTCTACTCAAGTTGGGTACATTTTACAGCCATCAGTGAGTTAGAAATAGGCTG
 >chr20:19615498-19615757 - E=1e-144 p=4e-02
TGTCTATGACATAGCATGCACAAATATGAAAAATGTAGTCAAATATCTTTTTCCTAAACATACGCACTGTGAAGATAATTAAATTTCTT

CTATCTTGGTTGGATGGTGTTTTATTTGTTGATTGGTTTTTAAATAAAATTTTTGCGGTATCATTTATAAGCAGTG|AAATT|CCTTCT
                                                                             >chr20:196
AGAGTGTGGAAAAAATTCTTTGGTTTGATGCCTTCTTACCTGTGCTCCAGTTTGTCACCTCTATGCAAAAAAAAAATGATTTGGCTAAG
30741-19630854 + E=2e-47
GGTAGGCCCTGAA|GAGGCGGCTGTAAGTTTAATACAATTCAG
```
